# The Viral Knock: Ameliorating Cancer Treatment with Oncolytic Newcastle Disease Virus

**DOI:** 10.3390/life13081626

**Published:** 2023-07-26

**Authors:** Upasana Pathak, Ramprasad B. Pal, Nagesh Malik

**Affiliations:** 1 Sir H.N. Medical Research Society, Sir H.N. Reliance Foundation Hospital and Research Centre, Mumbai 400004, Maharashtra, India; 2 Vivekanand Education Society’s College of Arts, Science and Commerce, Chembur, Mumbai 400071, Maharashtra, India

**Keywords:** cancer treatment, oncolytic viruses, Newcastle disease virus

## Abstract

The prospect of cancer treatment has drastically transformed over the last four decades. The side effects caused by the traditional methods of cancer treatment like surgery, chemotherapy, and radiotherapy through the years highlight the prospect for a novel, complementary, and alternative cancer therapy. Oncolytic virotherapy is an evolving treatment modality that utilizes oncolytic viruses (OVs) to selectively attack cancer cells by direct lysis and can also elicit a strong anti-cancer immune response. Newcastle disease virus (NDV) provides a very high safety profile compared to other oncolytic viruses. Extensive research worldwide concentrates on experimenting with and better understanding the underlying mechanisms by which oncolytic NDV can be effectively applied to intercept cancer. This review encapsulates the potential of NDV to be explored as an oncolytic agent and discusses current preclinical and clinical research scenarios involving various NDV strains.

## 1. Introduction

Time and again, viruses have changed the course of human history, affected the fabric of life for millions across the globe, led to a downfall in economies, and induced life changes that no one could have predicted. Even though they are feared as disease-causing agents, viruses also work wonders, from shaping evolution since the very beginning to, now, offering hope for curing cancer [1,2,3].

Oncolytic virotherapy is an innovative and promising approach to cancer treatment [4]. It utilizes wild-type or genetically modified OVs, which can kill cancer cells while discriminating against healthy cells [5,6]. The viral agents can be small RNA viruses, which encode only a few genes and have a short replication time, or large DNA viruses such as adenovirus, vaccinia virus, or herpesvirus, which encode over 250 different viral genes and thus allow a greater scope for genetic manipulation [7]. As cancer cells undergo many genetic alterations, the need of the hour is to identify and thoroughly research viruses with different oncolysis mechanisms as a single virus cannot be eligible to treat all cancers similarly [8]. The selection of an oncolytic virus is based on the stability of the virus, its therapeutic index, degree of pathogenicity, and whether it can invoke an immune response that can be directed against tumor cells [9,10].

Most cells lose components that are critical for innate antiviral defense during the transformation into cancer cells, making them more vulnerable to various virus strains than non-transformed cells. Cancer cells are less receptive to the stimulation of the antiviral response by interferons (IFNs) or tumor necrosis factor (TNF) [8]. The main mechanisms for productive viral infection and replication in host cancer cells are (i) receptor-mediated uptake due to the overexpression of virus entry-specific receptors on cancer cells and (ii) the adaptation of the virus to the host cellular oncogenic signaling pathways for viral replication [10]. Also, with the advancements in recombinant DNA technology and microRNA functions, the idea of “designer viruses” seems favorable [11]. Currently, viruses that are genetically engineered to express suicidal genes, a process known as gene-directed enzyme prodrug therapy (GDEPT), and immunostimulatory agents that are tumor-specific, evoke inflammatory responses, and have limited or no potential to replicate in non-cancerous cells are alluring anti-cancer agents [12,13,14].

## 2. NDV-Mediated Oncolysis

NDV is a single-stranded, non-segmented, negative-sense RNA genome avian virus called avian paramyxovirus type 1 (APMV-1). It largely impacts the poultry industry and is of great economic importance. The genomic RNA of NDV consists of six genes encoding at least eight proteins, namely, phosphoprotein (P), nucleoprotein (NP), large polymerase protein (L), fusion protein (F), hemagglutinin-neuraminidase (HN), matrix protein (M), and two nonstructural proteins, V and W [15]. In chicken cells, V protein is responsible for inhibiting the interferon response and apoptosis, but this escape mechanism function is observed only in birds and not in mammalian cells. Therefore, V protein is attributed as a reason for the reduced NDV host range. This is considered one of the main reasons that NDV is not virulent in normal mammalian cells [16]. NDV strains can be classified and designated depending on the degree of virulence and type of pathogenicity it causes in birds. In addition, the lentogenic pathotype of NDV mildly affects the respiratory system and is found to be the least virulent compared to the other two pathotypes, mesogenic and velogenic. There has been no reported death in poultry caused by a lentogenic NDV infection. Poultry is vaccinated mostly with lentogenic NDV strains (due to low virulence) like the Ulster, NDV Hitchner-B1 (HB1), and LaSota strains [17]. Lentogenic strains of NDV have been vastly researched for their application in oncolysis. Moderate virulence is observed in poultry due to infection with the mesogenic pathotype, MTH-68/H, and PV701 mesogenic strains have an established oncolytic potential. Velogenic NDV strains are highly pathogenic, causing death in all birds they infect [18,19]. These strains can be further subcategorized into viscerotropic and neurotropic velogenic strains. NDV strains are also classified, based on their oncolytic mechanism, as lytic and non-lytic for mammalian cells. Virulence in NDV is due to the fusion protein consisting of a (multi) basic cleavage site. The NDV HN and F proteins facilitate the infected cell’s fusion with the surrounding cells, resulting in the formation of syncytia and oncolysis. Velogenic NDV strains are lytic strains that cause lysis of host cancer cells, by producing infectious progeny virions and syncytial formation. The mesogenic strains like MTH-68/H, PV701, and Anhinga, defined as lytic strains, are also promising NDV oncolytic virus strains due to the induction of strong syncytia formation in vitro. On the other hand, non-lytic NDV disrupts normal host cell metabolism [20,21,22,23].

Various cell death pathways induced by NDV are discussed below.

### 2.1. Apoptosis

Apoptosis in cancer cells is delayed due to their dysregulated IFN response and defective STAT1 signaling pathway. These cancer cells are unaffected by antiviral enzymes, which enable viral replication and stimulate more infection by further virion production [24]. Velogenic NDVs induce a very robust apoptotic response that occurs early during infection. For example, NDV AF2240 induces apoptosis, possibly during virus binding or at the stage of fusion with the cell membrane [25,26]. Moreover, NDV exerts both intrinsic and extrinsic apoptosis pathways for oncolysis, irrespective of p53 activity [25]. A preliminary investigation by Fabian et al. showed that p53 expression did not affect U373 human glioblastoma sensitivity to NDV MTH-68/H [27]. A 2016 study showed that a velogenic NDV infection reduced hypoxia-induced HIF-1a accumulation, which controls the pro-survival protein found in hypoxic cancer cells. This NDV-induced downregulation of HIF-1a was post-translational and independent of p53 [28].

The Bcl-2 protein family consists of proteins that demonstrate anti- and pro-apoptotic functions, which control the intrinsic pathway and regulate different mitochondrial checkpoints [29]. The Bcl-2 family of pro-apoptotic proteins shows the presence of BH1- and BH3-like domains, which are also found in HN, F, M, and L NDV proteins. These domains promote the viral interaction with the Bcl-2 family proteins, leading to the functional modulation of these proteins [30,31]. Another study demonstrated that NDV infection resulted in Bax protein conformation change. This results in the transfer of Bax from the cytoplasm to the mitochondria, and cytochrome c is released into the cytoplasm. However, Bcl-2 protein expression levels remained unchanged by NDV infection. The Bcl-2 and Bax interplay modulates apoptosis in most of the cells infected by NDV, but this has not been conclusively determined for all the cell lines and viral strains from the in vitro studies performed. In one study, the expression levels of endogenous Bcl-2 and Bax were assessed in HeLa cells infected with NDV AF2240, but no significant change was observed in their level of expression [30]. Another study determined that Bax absence in human colon carcinoma HCT116 deferred but did not terminate the apoptosis activation process [32]. The infection of the MCF-7 cell line with the velogenic NDV AF2240 strain inactivated by UV stimulated apoptosis via the intrinsic pathway and activated caspase-8, indicating that NDV induces apoptosis in MCF-7 cells by both apoptosis pathways [26,33].

**Figure 1:**
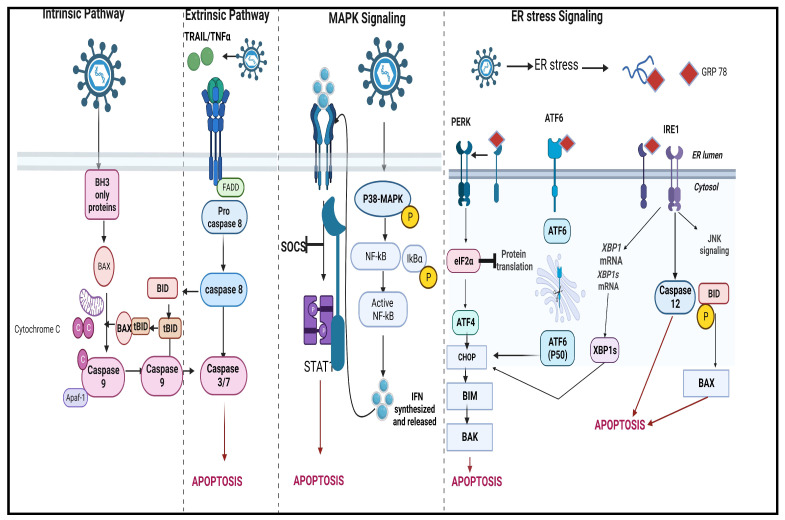
A brief description of NDV-induced cell death pathway: The intrinsic apoptosis pathway recruits bax to the mitochondrial membrane surface, by which it undergoes conformational change and initiates pore formation, and the release of cytochrome c. NDV infection leads to the release of the cytokines TRAIL and TNF-α, which then trigger the extrinsic pathway. The death receptors stimulate FADD. This activates caspase-8, which cleaves Bid into active tBid. The tBid along with the Bax protein stimulates MOMP. Caspase-8, activated by FADD, stimulates caspase-3/7. Both pathways lead to apoptosome formation via the interaction with Apaf-1 and cytochrome c. This apoptosome then activates caspase-9, which finally results in the activation of caspase-3/7. p38 MAPK pathway NDV infection phosphorylates MAPK and the inhibitor of nuclear factor κB alpha (IκBα). IκBα is degraded and releases active nuclear factor kappa B (NF-κB), which is transferred to the cell nucleus, and IFN-β is synthesized. The increased production of IFN-β phosphorylates STAT1 and degrades suppressor of cytokine signaling (SOCS) proteins which are the inhibitors of JAK/STAT signaling pathway. UPR triggers protein kinase RNA-like endoplasmic reticulum (ER) kinase (PERK)-activating transcription factor (ATF) 6 and inositol-requiring enzyme (IRE) 1. PERK activation results in phosphorylation of eukaryotic translation initiation factor 2α (eIF2α), which activates the expression of multiple transcription factors, like activation transcription factor 4 (ATF4). ATF4 induces the expression of the pro-apoptotic CCAAT/enhancer-binding protein-homologous protein (CHOP). Activated IRE1α cuts intron in the mRNA precursor of X-box binding protein 1 (XBP1), which is induced by ATF6. The transcription factor XBP1s initiates transcription of CHOP genes and stimulates the endoplasmic-reticulum-associated degradation. The UPR activates cell death via apoptosis by stimulating CHOP, JNKs, bax, and caspase [43,44,45]. (Figure created using BioRender).

In the extrinsic pathway, TNFα, FasL, and TRAIL are stimulated due to NDV infection and bind to their respective cell surface receptor proteins (Figure 1). This results in the oligomerization of receptors, and adaptor molecules are released to form the death-inducing signaling complex (DISC). Eventually, caspase-8 is stimulated, which activates the execution pathway [34,35]. Iraqi oncolytic virulent NDV was tested for its ability to induce apoptosis in vitro in several cell lines, like murine mammary adenocarcinoma cell line (AMN3) and human rhabdomyosarcoma (RD), via the intrinsic pathway, although a low level of caspase-8 was detected. At the same time, the female mice implanted with mammary adenocarcinoma cancer cells (AM3) and injected with NDV were checked for caspase-3, caspase-8, and caspase-9 expression. The results showed a significant expression of caspase-9 as well as caspase-8 and established that NDV induced an intrinsic apoptotic pathway in vivo along with an extrinsic pathway. The extrinsic pathway was reported to occur late in NDV-infected cells, which showed that the expression of surface bound and soluble TRAIL could be as late as 48 h post-infection in some cell lines like SH-SY5Y neuroblastoma cells, Hela, HepG2, and MCF7 [25].

NDV infection also triggers endoplasmic reticulum (ER) stress and p38 MAP kinase signaling pathways, ultimately activating and augmenting apoptosis [27,36]. 

According to a report that studied NDV-induced apoptosis mechanisms, NDV infection stimulated all three groups of the unfolded protein response (UPR) (PERK-eIF2α, ATF6, and IRE1α) and initiated apoptosis in CEF and DF-1 as well as human cancer cell lines like HeLa, HN13, Cal2, H1299, A549, HepG2, and Huh7 [37]. UPR is a conserved pathway in eukaryotic cells that promotes cell survival during ER stress. PERK/PKR-eIF2α signaling enhanced NDV proliferation and supported the translation of viral proteins, slashing the host translation components. IRE1α-XBP1 pathway also promoted virus protein modification, folding, assembly, etc. The activation of JNK by UPR led to apoptosis and triggered the secretion of cytokines [38,39]. However, ER stress can lead to cell death not only by apoptosis (intrinsic and/or extrinsic) but also by autophagy [37,39,40].

p38 MAPK is stimulated by stress and mitogens; p38 signaling is involved in cellular responses like cell differentiation, development, death, inflammation, senescence, and tumorigenesis [41]. Bian et al. demonstrated for the first time the involvement of the MAPK pathway in the NDV-induced infection of the A549 cell line, and the silencing of the p38 MAPK pathway lowered NDV-induced cell death [42].

### 2.2. Necroptosis

Necrosis also contributes to the NDV cytopathic effect. NDV infection leads to the expression of HN and F proteins on the host cell surface, further enhancing the formation of cell syncytia, cell fusion, and the destruction of syncytia, which ultimately triggers necrosis [46,47].

Cell necroptosis is stimulated via the cellular toll-like receptor (TLR) and the TNF family in the absence of caspase-8 [48]. Necrosis causes unprogrammed damaging changes in tissue and can also occur due to the stimulation of various other modes of cell death like necroptosis (RIP kinase-dependent), parthanatos (PARP-dependent) pathways, etc. [23,49,50]. A study by Koks et al. proved that NDV HB1 had an oncolytic effect on the GL261 glioma cell line as well as on a mouse model and induced immunogenic cell death via necroptosis. It was observed that caspase inhibition had no significant effect on immunogenic cytolysis, but the treatment of cells with necrostatin-1 reduced the cell death checked at 24 and 96 h post-infection [51]. On the contrary, a study reports NDV infection in HeLa cells inhibited necroptosis by activating NF-κB, which triggers the synthesis of TNF α and TRAIL, leading to the activation of caspase-8. Caspase-8 cleaves RIP1, although some amount of RIP1 remains intact and phosphorylates MLKL (necroptosis hallmark) to phosphor-MLKL and transfers it to stress granules by binding with RIP1, thus inhibiting MLKL transfer to the plasma membrane, which would culminate in necroptosis [49].

### 2.3. Autophagy

Autophagy, also known as type 2 programmed cell death, is a catabolic process that eliminates accumulated proteins, impaired organelles, and pathogens to maintain cellular homeostasis [52]. A double-membraned autophagosome that engulfs cytoplasm and cytoplasmic organelles is the hallmark of autophagy [53,54]. Viruses either suppress autophagy for their survival or utilize this pathway such that it aids replication [23,55]. NDV HN and F proteins induce autophagy, syncytia formation, and, consequently, oncolysis. NDV exploits autophagy (autophagy prolongs the cancer cell life cycle as these cells use autophagic components to meet their high metabolic requirements) for its replication in the early stages of cancer cell infection. Thus, autophagy constrains apoptosis, supports NDV replication in cancer cells, and stimulates the immune response to ultimately kill the cancer cells [56,57,58]. Autophagy induction in the immunogenic cell death of cancer cells is triggered due to the recognition of pathogen-associated molecular pattern molecules (PAMPS), damage-associated molecular patterns (DAMPS), and other cellular stress signals released in response to NDV infection. NDV, in combination with autophagy modulators, enhances NDV anti-cancer activity [59,60,61]. Recombinant NDV-expressing rabies virus glycoprotein (rL-RVG) and LaSota induced stomach adenocarcinoma cell death via autophagy along with the activation of the ER stress response and apoptosis [62].

### 2.4. Immune Response

**Figure 2:**
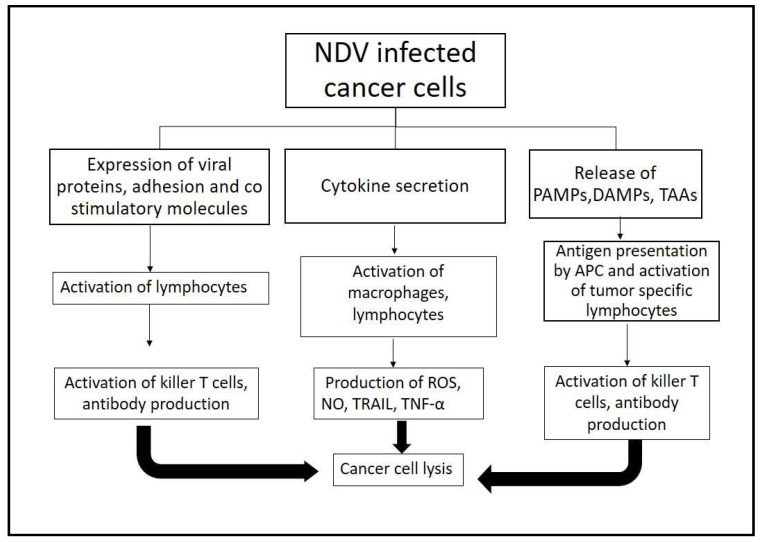
A summary of NDV-mediated immune response. The immune stimulation response includes the enhanced expression of adhesion and costimulatory molecules; viral glycoproteins on the infected cancer cell surface; and the secretion of proinflammatory cytokines, which activate lymphocytes and macrophages, ultimately producing effectors like ROS, TRAIL, and TNF α. Direct cancer cell lysis due to NDV infection via ICD leads to release of PAMPS, DAMPS, and TAA, which activate APCs and tumor-specific lymphocytes, resulting in T cell activation and antibody production due to B cell activation [17,70].

The immunostimulatory effect of NDV is also responsible for its oncolytic potential. Cytokines such as interferons, TRAIL, and TNF are stimulated by NDV, and these effector cytokines in turn activate macrophages, natural killer (NK cells), and dendritic cells and sensitize T-cells [63]. The inflammatory milieu induced by NDV leads to the stimulation of innate effector cells and adaptive immune cells that enable anti-cancer immunity (as shown in Figure 2). Cancer cells infected by NDV result in enhanced cancer cell immunogenicity [35,64]. NDV replication is also suppressed in some cancer cells after prior treatment with type 1 IFN. Therefore, it is crucial to understand the exact stage of type I IFN induction to achieve a steady virus replication rate and stimulate innate immune response to augment adaptive immunity [21,65].

It has been observed that the cellular cytotoxicity of PBMC towards cancer cells significantly increased post-incubation with NDV [66]. NDV activates the immune response in host cells, leading to the production of IFN-α, IFN-β, IFN-γ, IL-1, etc. It also induces the production of chemokines like RANTES and IFN-γ inducible protein 10 (IP-10). Additionally, the upregulation of ICAM-I0, LFA-3, and HLA-DR in cancer cell lines was detected. The interaction between viral HN glycoprotein and host cell sialic acid residue has been found to activate NK cells. In vitro bystander antitumor activity on human tumor cell monolayers is reported due to activated human NK cells (amplified antitumor activity) [18,21,67,68]. In one study, mice immunized with NDV LaSota resulted in decreased NDV replication due to the neutralizing antibodies produced. However, the anti-cancer activity was intact and can be attributed to an adaptive immune response directed towards NDV infected cancer cells and epitope spread [69].

## 3. Progress in Utilization of NDV Strains as Oncolytic Virus

### 3.1. Wild-Type and Vaccine Strains

Naturally, oncolytic NDV strains are being investigated in pre-clinical and clinical models for their oncolytic potential. LaSota is a lentogenic strain investigated for its potential to induce significant anti-cancer effects in MCF7 and Arabian and Middle Eastern breast cancer cell lines (AMJ13), mainly via the intrinsic apoptotic pathway, as the percentage of caspase-9 was found to be higher than that of caspase-8 [71]. The safety and efficacy of the wild-type NDV/HK84 strain when checked in vitro as well as in vivo in hepatocellular carcinoma showed type I IFN-dependent cytopathic effects. This strain showed a very low replication rate in normal mouse hepatocytes, making it a promising viral candidate for further investigations [72]. Another strain, HB1, induced apoptosis (via the intrinsic pathway) as well as autophagy in the cervical cancer model. Cancer cells exhibited increased cytochrome C, caspase-3, and ROS levels indicative of apoptosis. Also, microtubule-associated protein 1 light chain 3 (LC3) was upregulated, which confirmed autophagy [73]. Gamma-irradiated GBM cell line NCH 149, when infected with NDV Ulster, induced cytokine production, leading to cancer cell death [63]. Also, Ulster induced robust cytotoxic activity in the CT26 colon carcinoma model [74].

Christine Csatary was the first to report the mesogenic NDV strain Hertfordshire for its oncolytic potential and derived an attenuated strain named MTH-68/H, which means “More Than Hope 1968”. MTH-68/H induces significant cytotoxicity in cell lines such as MCF-7, PC12, DU-145, HCT116, A431, HT-29, PC3, and HeLa cells. MTH-68/H is the most effective IFN-α inducer and also stimulates NO [27,75].

NDV strain AF2240 is a velogenic Malaysian field outbreak isolate; extensive research is being carried out as it has been proven to have exceptional oncolytic potential. The immunogenicity of AF2240 studied by PBMC stimulation and macrophage infection was found to be significant, and the oncolytic ability of the strain was maintained even in the hypoxic tumor microenvironment. It exerts a dose-dependent cytotoxic effect on MCF-7, MDA-MB231, and HeLa cells. However, viral oncolysis was not very significant against cisplatin-resistant MCF-7 cells [26,33,66,76,77,78].

### 3.2. Genetic Manipulations

Reverse genetic engineering techniques for negative-strand RNA viruses introduced the possibility of genetically engineering NDV, thus amplifying and strengthening the oncolytic potential with no side effects involved in therapy [79].

Genetically engineered NDV F gene:

In NDV, the fusion protein cleavage site is a key protein for membrane fusion as well as virulence. A non-fusogenic NDV Hitchner B1 (NDV B1) was engineered using reverse genetics wherein the F protein cleavage site was modified with three extra arginine residues, resulting in a multibasic cleavage and activation site. This rNDV/F3aa provided a more effective, sustained NDV infection in host cells and has also proved to be an effective viral backbone for further genetic modifications [80]. A genetically modified NDV-B1/F3aa, along with a green fluorescent protein (GFP), was constructed using a DNA fragment GFP inserted between the P and M genes of pT7NDV/F3aa. The anti-cancer potential was tested in a mouse model bearing MKN-74 human gastric adenocarcinoma cells. The mouse models exhibited an oncolytic effect without any signs of toxicity, which could be easily detected by evaluating the infectivity by the GFP marker [81]. Another in vivo study revealed that following recombinant rNDV/F3aa L289A (which had an L289A mutation in the F gene) administration, the tumor tissue of hepatocellular carcinoma showed increased necrotic changes. However, healthy tissue adjacent to the cancer cells projected no hepatotoxicity, no inflammation, and no syncytium formation, which is characteristic of NDV infection [82].

Targeting tumor antigens:

Genetically engineered NDV-expressing tumor-associated antigens (TAAs) present an approach for using NDV as a vaccine vector. β-gal-specific CD8 T-cell epitope TPHPARIGL (minigal) cDNA was cloned between the NDV P/V and M genes of an NDV clone (NDV/F3aa). This model stimulated a targeted TAA-specific adaptive immune reaction leading to tumor regression in 60% of mice bearing murine colorectal carcinoma CT26 tumors. Furthermore, this antitumor efficacy was boosted by combination treatment with NDV-expressing IL-2, which showed complete tumor regression in 90% of the mice [79]. CD147 is a molecule that is upregulated on the cancer cell surface and enhances cancer progression, metastasis, and angiogenesis and regulates the tumor microenvironment [83]. Wei et al. in 2015 constructed NDV-expressing 18 HL (an antibody against CD147). Orthotopic HCC xenograft mice injected with rNDV-18HL exhibited significantly less intrahepatic metastasis and increased the survival rate in mice compared to those injected with control strain NDV Italien [84].

Modification using cytokines:

cDNA consisting of human IL-2 and TRAIL genes were inserted between the NDV HN and L genes to obtain rNDV. rNDV-human IL-2 along with TRAIL triggered T cell activity and upregulated the expression of apoptotic genes (caspase-8, caspase-9, caspase-3, FasL, and BAX) in melanoma and HCC tumor cell mice models via TRAIL [85]. Similarly, rNDV expressing macrophage inflammatory protein-3α (MIP-3α) was constructed by inserting MIP-3α cDNA between the P and M genes. MIP-3α is a specific chemokine for DCs that amplified anti-cancer immunogenic reactions in B16 murine melanoma and CT26 colon carcinoma mouse models. Both models showed enhanced DC activation and maturation compared to the wild-type NDV [86]. Researchers have also enhanced the antitumor effects of non-lytic NDV LX by engineering it to co-express IL15 and IL7. Both interleukins are involved in the activation and development of T cells. B16-F10 (murine melanoma) cells infected with this rNDV show increased IL15 and IL7 expression. These tumor cells were then administered as a vaccine, which elicited the activation and proliferation of T cells and NK cells. The infiltration of CD4+ and CD8+ T cells was observed in TME. CD8+ T induced significant tumor suppression and better survival in melanoma mouse models [87]. MEDI5395, a recombinant NDV constructed with a GM-CSF transgene, exhibited cancer-cell-specific replication and induced tumor regression in murine and patient-derived xenograft models. Immune activation in the TME was observed as there was an enhanced expression of IFNγ-inducible genes and T cell activation. The PD-L1 blockade further improved the oncolytic activity [88].

Enhancing Co-Stimulatory Signal for T-cell Activation:

CD278, or inducible T cell co-stimulator (ICOS), is an important co-stimulator in T cell proliferation and cytokine production that binds to a CD278 ligand expressed by B-cells, macrophages, and DCs [89]. Recombinant NDV LaSota was engineered to express the CD278 ligand by using the transgene inserted between the NDV P and M genes. This NDV-CD278L was administered intratumorally into bilateral flank B16-F10 mouse models, which enhanced CD278 expression in the tumor microenvironment (TME). The high infiltration of activated T cells and delayed tumor growth were also observed. NDV-CD278L, when combined with anti-CTLA-4 blockade, induced a significant increase in CD8+ T cells and the upregulation of CD278 and granzyme B. Additionally, this efficacy improved local as well as distant tumor regression in mouse models [90]. Similarly, OX40 plays a co-stimulatory role in T cell activation and survival. OX40-OX40L interaction facilitates the stimulation and proliferation of both CD4+ and CD8+ T cells. rNDV expressing murine OX40L (rNDV-mOX40L) induced an increase in tumor inhibition rate by 15.8% in the CT26 model compared to NDV-treated controls. The tumor site showed an increase in CD4+ and CD8+ T cells as well as IFN γ levels [91].

Vector for tumor suppressor genes:

The recombinant plasmids consisting of the human p53 gene and helper plasmids encoding NDV NP, P, and L proteins were constructed and transfected into BHK21 cells. rNDV-53 produced from the cell culture was then amplified for use. The rNDV-p53 administration in the hepatocellular carcinoma model showed more than a 5-fold reduction in tumor weight, and 75% of treated mice showed a 120-day survival rate. The treatment was nontoxic, as suggested by the normal serum profile of the treated mice [92]. Phosphatase and tensin homolog (PTEN) is a tumor suppressor gene that encodes for a phosphatase enzyme. A mutation in this gene is commonly seen in a wide range of cancers. The PTEN gene was inserted in NDV between the NP and P genes and between the P and M genes. These rNDV were tested on glioblastoma cell lines in vitro and on glioblastoma xenograft mice models. rNDV with the PTEN gene inserted between NP and P genes proved to be effective in enhancing cytopathic activity and syncytium formation in the glioblastoma cell line, and a significant reduction in tumor size was observed on day two of virotherapy [93]. This rNDV-PTEN was further administered in the orthotropic U87 MG glioblastoma mouse model. rNDV-PTEN successfully crossed the blood–brain barrier and overexpressed PTEN, resulting in the apoptosis of cancer cells [94].

Facilitating NDV spread:

Matrix metalloproteinase 8 (MMP-8) is involved in the breakdown of the extracellular matrix during reproduction, embryo development, and disease progressions like metastasis and also regulates neutrophil chemotaxis in vivo. The ECM acts like a barrier and hinders the spread of viruses in tumors. Recombinant NDV-expressing MMP8 (NDV-MMP8) was constructed, and intratumoral injection efficiently degraded ECM, enhanced virus spread, and induced tumor regression in liver cancer xenograft mouse models [95].

### 3.3. Combination Therapy with Oncolytic NDV

Existing treatment approaches or drugs are also being explored for synergistic action with NDV. For example, a phase 1, first-in-human, multicenter, open-label trial involving patients with breast, colorectal, hepatocellular carcinoma, head and neck squamous cell carcinoma, renal cell carcinoma, and non-small-cell lung cancer is evaluating the MEDI5395 strain in combination with durvalumab (durvalumab is an immune checkpoint inhibitor, which inhibits programmed death ligand 1 (PD-L1), binding to programmed death 1 (PD-1) and CD80). The trial’s primary objective is to determine the safety and tolerable dosage of MEDI5395 and durvalumab. A secondary goal is to identify the efficacy, immunogenicity, pharmacokinetics, and pharmacodynamics of MEDI5395 [96,97]. A prostate cancer patient with bone metastases underwent complete remission due to treatment with local hyperthermia (LHT) and vaccination with viral oncolysate-DC (VOL-DC) and systemic NDV [98]. A recent study highlighted the synergistic action of doxorubicin with the NDV Iraqi strain on plasmacytoma cells independent of the p53 pathway. NDV combined with rituximab also showed high cytotoxicity on plasmacytoma cells, independent of the p53. Another study aimed to explore the synergistic effect of genetically engineered NDV LaSota L289A expressing anti-CTLA4 along with radiotherapy (10 Gy) as well as the systemic administration of anti-CTLA4 +NDV L289A+ radiotherapy (10 Gy) in a B16-F10 melanoma mouse model. Both methods of utilizing anti-CTLA4 antibodies effectively induced CD8+ T cell infiltration in TME, sensitized the tumors to radiotherapy, and enhanced the effectiveness of radiotherapy [99].

Non-virulent LaSota NDV combined with 5-FU was evaluated by Shammari et al. for its cytotoxic effect on human Hep-2 (larynx carcinoma) and rhabdomyosarcoma (RD) cell lines. NDV showed synergistic action with 5-FU at low doses, and there were minor effects on normal cells [100]. A combination of nisin A at a low concentration of 20.00 μg/mL and a vero cell line adapted an NDV-enhanced apoptotic effect compared to the individual effect [101]. Vanadyl sulfate (mostly used in diabetes treatment) can stimulate a robust immune response and hence was explored for its anti-cancer applicability. NDV (5 × 10^7^ PFU) and vanadyl sulfate (40 mg/kg), when injected in syngeneic B16-F10 melanoma mice, stimulated an innate immune response characterized by macrophages and NK cells and led to tumor regression 48–96 h post-administration [102].

### 3.4. Improving Systemic Delivery

Inefficient systemic delivery to the target or off-target effects results regarding the rapid clearance of the virus and exhibits a reduced oncolytic effect. Several attempts are being made to overcome these drawbacks concerning the virus delivery system.

A recent study demonstrated that the NDV LaSota virus when encapsulated in thiolated chitosan nanoparticles functionalized with hyaluronic acid bound to CD44 enabled the immune protection of the virus by masking and escalated the bioavailability of the virus in cervical cancer cells [103]. Temozolomide (TMZ) is an anti-cancer drug most commonly used to treat GBM patients. A study explored the TMZ-loaded Poly(lactic-co-glycolic acid) (PLGA) nanoparticles (NPs) along with the AMHA1-attenuated strain (Iraqi strain) of NDV as an alternative therapeutic. This synergistically enhanced the anti-cancer activity on the cerebral glioblastoma multiforme (AMGM5 cell lines). It was therapeutically effective, biocompatible, and safe. The combination of NDV and TMZ-PLGA-NPs definitely has future clinical use in cancer therapy [104].

Studies utilizing mesenchymal stem cells (MSCs) for oncolytic NDV delivery provide an effective Trojan Horse approach for better tumor tropism and cancer cell death. MSCs infected with NDV (MTH-68/H) delivered the virus to glioma cell lines (A172 and U87) as well as primary glioma stem cells (GSCs) that were derived from GBM tumors, and the cytotoxicity, survival, and renewal of GSCs due to NDV-infected MSCs were investigated. Glioma cells were sensitized to NDV infection by MSCs and induced dose-dependent, TRAIL-mediated killing in glioma cells and GSCs. The infected MSCs increased γ-radiation sensitivity in GSCs [105].

Another study obtained MSCs from the bone marrow of C57BL mice and studied the effect of MSCs encumbered with oncolytic NDV on splenic T cells and cytokine immune responses. The results demonstrate that oncolytic NDV delivered by MSCs effectively reduced cancer cell proliferation, induced CD8+ T cell cytolysis response, and upregulated caspase-9 expression [106].

## 4. Future Considerations and Conclusions

The oncolytic action of wild-type or attenuated NDV is poorly understood, leading to a lack of progress in preclinical and clinical trials. Wild-type NDV strains show specificity only for a few cancer types, while many cancer cells show resistance to NDV replication. Therefore, a large number of wild-type strains that aggressively replicate in tumor cells will have to be screened and genetically modified for the highest specificity and safety [107]. Defined clinical guidelines for systemic delivery of oncolytic viruses are needed as the delivery route differs based on the tumor location, and any uncontrolled biosafety negligence can have an adverse effect on the patient [108]. Incessant endeavors are also required for developing an effective delivery system for NDV transport to cancer cells. Nanoparticles, liposomes, or other synthetic carriers can offer a promising role in shielding NDV until the virus is in circulation, enhancing virus accumulation in the cancer cells and improving overall therapeutic efficacy [109]. The immunosuppression of NDV poses as one of the major hurdles, such as a low therapeutic index leading to the need for high dosage administration [110]. Furthermore, pre-clinical studies involving 3D cancer cell models are required to delineate NDV behavior (penetration, replication, and spread) in vivo [111]. Optimum virus dosage, sustainable delivery agent, route, and appropriate genetic modifications can contribute to overcoming both specific and nonspecific host immune reactions.

Although various types of cancer treatment modalities, such as surgery, radiation therapy, and/or systemic therapy, which include chemotherapy, hormonal therapy, immunotherapy, and targeted therapy, exist, none of these approaches are a failsafe for combating metastatic relapse [112]. The cancer treatment arena is continuously transforming with time, and integrating NDV oncolytic virotherapy can revolutionize the era of modern oncotherapeutics.

## Data Availability

Data is contained within the article.
